# Infrared imaging and intravascular laser irradiation of blood therapy for glycemic control in type 2 diabetes mellitus: a technical note

**DOI:** 10.3389/fmedt.2025.1701628

**Published:** 2026-02-03

**Authors:** Philype Antonio Calazans Candido, Gabriel Carneiro Brioschi, Marcos Leal Brioschi, Kathleen Melchior Altruda

**Affiliations:** 1 Brazilian Medical Thermology Association (ABRATERM), Medical Thermology and Thermography Specialization, Faculty of Medicine, University of São Paulo (HCFMUSP), São Paulo, Brazil; 2 Kent State University, Kent, OH, United States; 3 American Academy of Thermology (ATT), Greenville, SC, United States; 4 University of São Paulo (USP), Anhembi Morumbi University (UAM), Brazilian Association of Thermography (ABRATERM), São Paulo, Brazil

**Keywords:** diabetes mellitus, laser therapy, low-level light therapy, thermography, type 2

## Abstract

This technical note presents a standardized method that integrates intravascular laser irradiation of blood (ILIB) with infrared thermography to visualize real-time physiological responses during treatment. One adult male with long-standing type 2 diabetes underwent ten ILIB sessions using a 660 nm transcutaneous laser applied over the left radial artery. Infrared thermography captured temperature changes in abdominal regions associated with metabolic function. Across sessions, an average temperature increase of approximately 2.1 °C was observed in the hepatic and epigastric areas. Laboratory values showed reductions in fasting glucose (−29%) and HbA1c (−14%), while C-peptide remained stable. Because this is a single-patient technical demonstration, the findings cannot be interpreted as evidence of efficacy. Instead, the report illustrates the feasibility and potential utility of combining ILIB with thermal imaging for methodological development in future controlled studies.

## 1 Introduction

Photobiomodulation delivered through intravascular laser irradiation of blood (ILIB) has been explored for its potential effects on microcirculation and cellular bioenergetics (1, 2). Infrared thermography offers a non-invasive means of monitoring superficial temperature distributions, which may indirectly reflect physiological changes related to blood flow and metabolic activity (3). When combined, ILIB and thermal imaging provide an opportunity to document immediate physiological responses that occur during laser application.

The aim of this technical note is to present a reproducible protocol integrating ILIB with infrared thermography and to illustrate its feasibility in a representative patient. This work focuses solely on methodological demonstration rather than clinical evaluation.

## 2 Materials and methods

### 2.1 Study design

This report describes a technical demonstration using one adult patient. The goal was to document the combined use of ILIB and thermography rather than assess therapeutic outcomes.

### 2.2 Patient information

A 62-year-old male with a 30-year history of type 2 diabetes participated. Baseline laboratory results included fasting glucose 331 mg/dL, HbA1c 8.5%, and C-peptide 0.83 ng/mL. His antidiabetic therapy (NPH insulin plus oral medications) remained unchanged throughout the demonstration period.

### 2.3 ILIB intervention protocol

**Figure 1:**
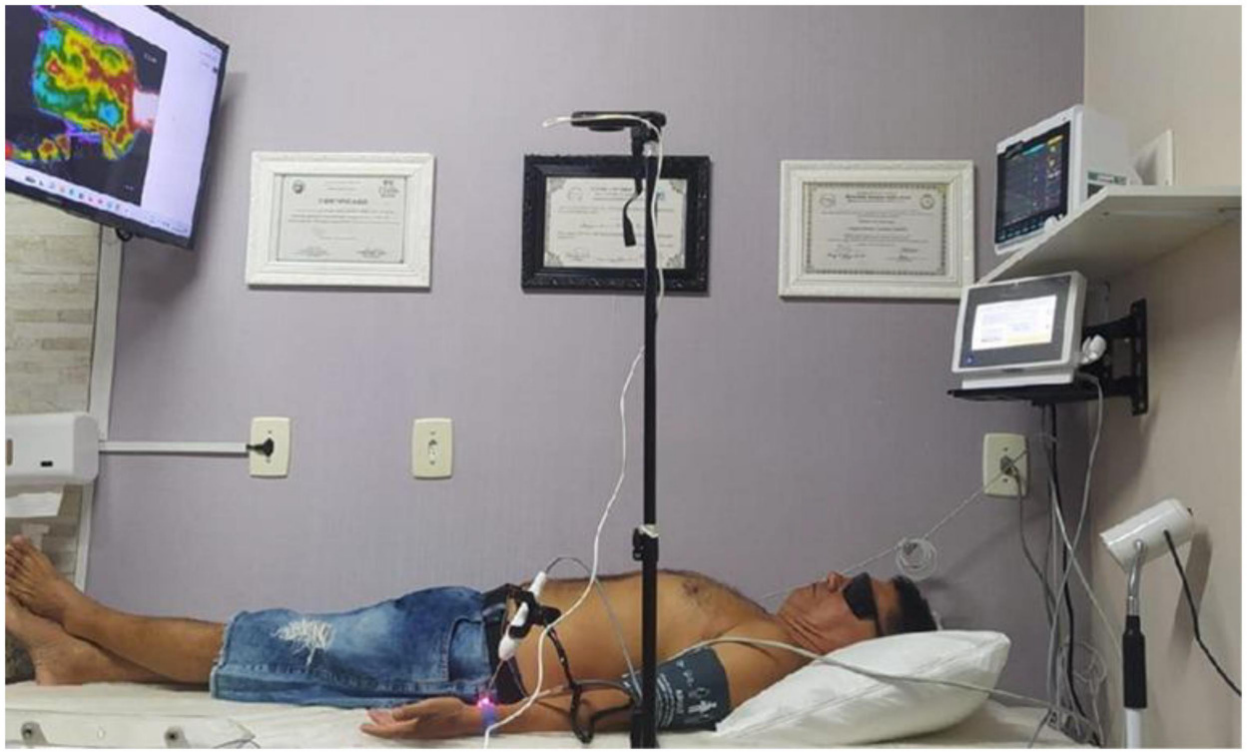
ILIB setup and imaging configuration. The figure shows the patient positioned supine with the 660 nm ILIB probe aligned over the left radial artery. A FLIR C5 infrared camera, positioned 1.3 m above the thoracoabdominal region, captures thermal data throughout the session. The image illustrates the standardized acquisition environment used across all examinations.

ILIB was performed using a 660 nm ± 10 nm InGaAlP red laser (100 mW ± 20%). The procedural setup, including probe placement and imaging configuration, is illustrated in Figure 1. The probe was positioned transcutaneously over the left radial artery. Each session lasted 30 min, delivered twice weekly for ten sessions. The patient rested for 15 min before imaging. Protective eyewear was used throughout all procedures.

### 2.4 Imaging procedure

Infrared thermographic imaging was performed using a FLIR C5 camera (160 × 120 pixels; 8–14 µm spectral range) placed 1.3 m above the thoracoabdominal region. Images were collected at baseline and at 10, 20, and 30 min during each ILIB session. Regions of interest (ROI) included the right hypochondrium, the epigastrium, and the periumbilical area.

### 2.5 Environmental conditions and monitoring

Room temperature was controlled at 23 °C with 50% relative humidity. Airflow remained below 0.2 m/s. The patient was positioned supine on a gurney. Vital signs—blood pressure, heart rate, respiratory rate, and oxygen saturation—were recorded every 10 min using a multiparameter monitor.

### 2.6 Data recording

Temperature values from the ROIs were extracted using the camera's analysis tools. Because this is a technical demonstration, thermal patterns were interpreted descriptively without quantitative validation or inter-observer comparison.

## 3 Results

**Figure 2:**
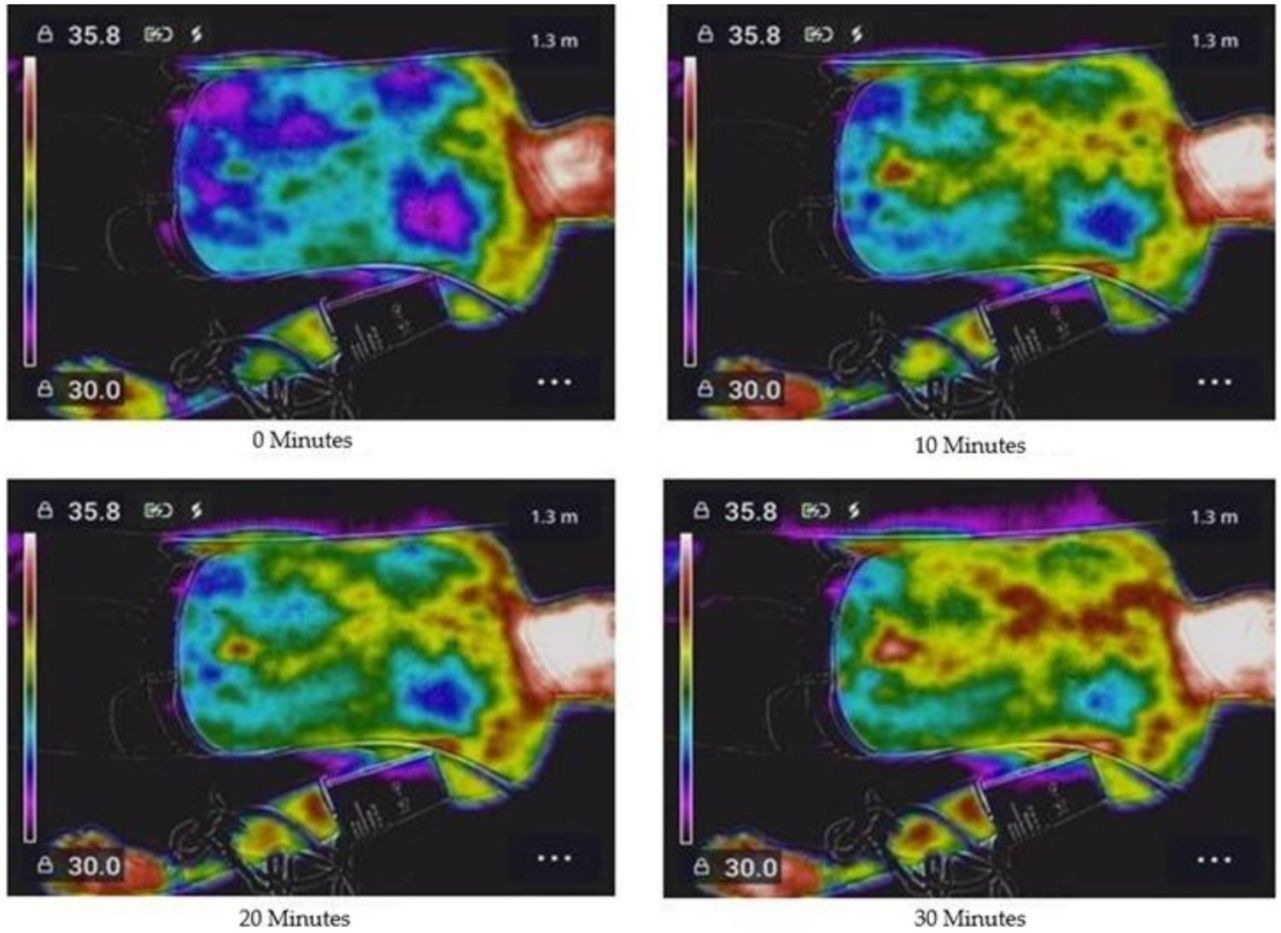
Sequential thermographic images acquired during ILIB. Thermal images obtained at 0, 10, 20, and 30 min demonstrate a progressive increase in abdominal surface temperature during ILIB application. Regions of interest include hepatic, epigastric, and periumbilical areas. Environmental conditions were controlled to minimize interference.

**Figure 3:**
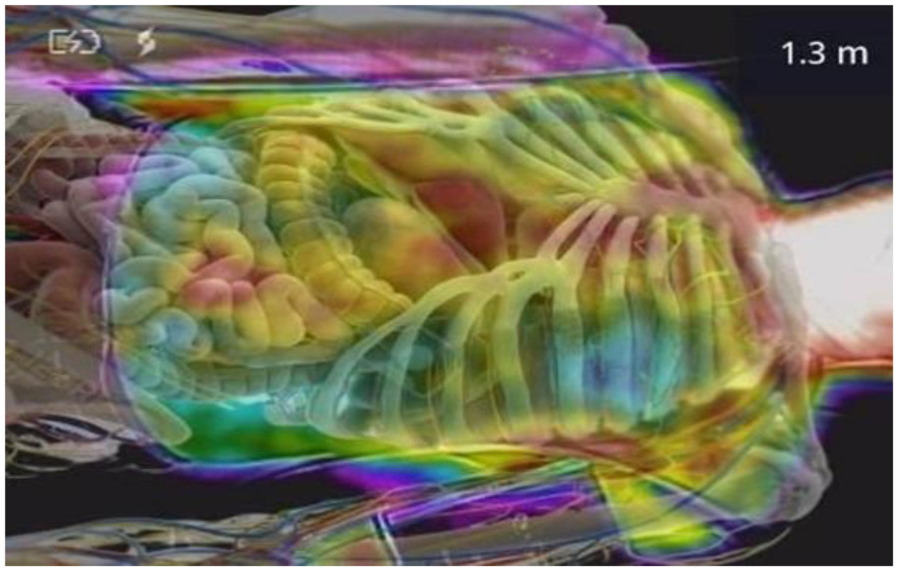
Composite thermal map highlighting regions of hyperthermic response. A merged representation of thermal data from multiple sessions showing consistent zones of increased temperature corresponding anatomically to hepatic and gastrointestinal perfusion.

Thermal imaging consistently demonstrated progressive increases in abdominal temperature during each ILIB session (Figure 2). The hepatic and epigastric ROIs displayed a mean temperature rise of approximately 2.1 °C between baseline and 30 min. These patterns were reproducible across the ten sessions and are summarized visually in the composite thermal map shown in Figure 3.

Vital signs remained within normal ranges throughout. Minor fluctuations in blood pressure were observed but are likely attributable to situational responses, including possible white-coat effect.

Following the ten-session protocol, fasting glucose decreased from 331 to 234 mg/dL (−29%), and HbA1c decreased from 8.5% to 7.3% (−14%). C-peptide levels changed minimally (−3%). These laboratory values are presented in Table 1 for clarity. Three nocturnal hypoglycemic episodes occurred and were resolved with carbohydrate intake. These metabolic results are presented solely as observational findings and cannot be attributed to ILIB.

**Table 1:** Laboratory values before and after ten ILIB sessions, including percentage changes. The table summarizes fasting glucose, HbA1c, and C-peptide values used for descriptive comparison.

Date	Fasting glucose	Change (%)	HBA1C	Change (%)	C-peptide	Change (%)
17/10/2023	331 mg/dL	–	8.5%	–	0.83	–
04/12/2023	234 mg/dL	−29%	7.3%	−14%	0.80	−3.6%

## 4 Discussion

This technical demonstration illustrates that infrared thermography can be paired with ILIB to visualize physiological changes during treatment. The temperature increases observed over the abdominal regions align with previous descriptions of photobiomodulation-associated changes in microcirculation (4, 5). Comparative studies of photobiomodulation and ILIB protocols have also reported physiological changes detectable through imaging or biochemical markers, supporting the rationale for combining ILIB with real-time monitoring tools (6). However, thermography is an indirect assessment tool susceptible to multiple confounding influences, including environmental factors, emotional state, and autonomic responses.

Metabolic changes observed in this patient must be interpreted carefully. Single-subject observations cannot distinguish true intervention effects from natural glycemic variability, medication adherence, dietary fluctuations, or regression to the mean. Previous ILIB literature emphasizes the need for well-controlled trials before drawing clinical conclusions (1, 2). Percentage changes are reported for descriptive clarity but do not imply statistical significance.

Mechanistic explanations—such as ATP modulation, nitric oxide release, reactive oxygen species regulation, or improved endothelial function—were not evaluated in this demonstration and remain hypothetical. Future investigations should include biochemical assays, vascular imaging, and continuous glucose monitoring to evaluate physiological pathways more thoroughly. This technical demonstration illustrates that infrared thermography can be paired with ILIB to visualize physiological changes during treatment. The temperature increases observed over the abdominal regions align with prior descriptions of photobiomodulation-associated changes in microcirculation (4, 5). However, thermography is an indirect assessment tool susceptible to multiple confounding influences, including environmental factors, emotional state, and autonomic responses.

Metabolic changes observed in this patient must be interpreted carefully. Single-subject observations cannot distinguish true intervention effects from natural glycemic variability, medication adherence, dietary fluctuations, or regression to the mean. Previous ILIB literature emphasizes the need for well-controlled trials before drawing clinical conclusions (1, 2). Percentage changes are reported for descriptive clarity but do not imply statistical significance.

Mechanistic explanations—such as ATP modulation, nitric oxide release, reactive oxygen species regulation, or improved endothelial function—were not evaluated in this demonstration and remain hypothetical. Future investigations should include biochemical assays, vascular imaging, and continuous glucose monitoring to evaluate physiological pathways more thoroughly.

## 5 Limitations

This technical note carries inherent limitations. As a single-case demonstration, the findings cannot be generalized or interpreted as indicators of clinical benefit. The absence of a control condition restricts the ability to distinguish ILIB-related responses from natural physiological variability. Dietary intake, physical activity, body weight, and medication adherence were not monitored, although these factors are known to influence glycemic and metabolic parameters.

Thermographic assessment was qualitative and lacked inter-observer reliability testing or standardized quantitative ROI analysis. Thermal measurements are sensitive to environmental and physiological noise, which may influence interpretation. Additionally, no complementary physiological measurements—such as Doppler ultrasound, biochemical assays, or continuous glucose monitoring—were performed. Consequently, mechanistic explanations remain speculative.

Blood pressure variations may reflect situational or white-coat effects rather than treatment-induced changes. Finally, the ILIB parameters described here should not be considered optimized for clinical use. Larger controlled studies with standardized imaging protocols and comprehensive physiological monitoring are required to evaluate reproducibility, safety, and clinical relevance.

## 6 Conclusions

This technical note demonstrates an innovative and feasible protocol that integrates intravascular laser irradiation of blood (ILIB) with infrared thermography to monitor physiological responses in real time. The combination of these two techniques provides a standardized and reproducible workflow capable of capturing dynamic thermal patterns associated with metabolic activity. Importantly, this approach introduces a novel imaging-based framework for evaluating systemic photobiomodulation procedures, offering a level of visualization that has not been previously reported in ILIB applications.

Although this single-patient demonstration cannot support clinical inference, the protocol establishes a methodological foundation that can be directly incorporated into future controlled studies. By enabling objective thermal monitoring during ILIB, the proposed model has the potential to improve procedural standardization, support mechanistic investigation, and guide the development of optimized ILIB parameters for metabolic and microcirculatory research. This work expands the technical toolbox available to the photobiomodulation field and may contribute to more rigorous experimental designs in upcoming clinical investigations.

## Data Availability

The raw data supporting the conclusions of this article will be made available by the authors, without undue reservation.

## References

[1] Karu TI. Low-power laser therapy. In: Vo-Dinh T, editor. Biomedical Photonics Handbook. Boca Raton: CRC Press (2003). Chapter 48. p. 1–25.

[2] KazemiKhoo N Ansari F. Blue or red: which intravascular laser light has more effects in diabetic patients? Lasers Med Sci. (2015) 30(1):363–6. 10.1007/s10103-014-1672-7 25304768

[3] Ring EFJ Ammer K. Infrared thermal imaging in medicine. Physiol Meas. (2012) 33(3):R33–46. 10.1088/0967-3334/33/3/R33 22370242

[4] Herrera MA Ribas AP da Costa PE Baptista MS. Red-light photons on skin cells and the mechanism of photobiomodulation. Front Photonics. (2024) 5:1460722. 10.3389/fphot.2024.1460722

[5] Karu TI. Molecular mechanisms of low-level laser therapy. Dokl Akad Nauk SSSR. (1986) 291(5):1245–9. 3542462

[6] Hamblin MR. Mechanisms and applications of the anti-inflammatory effects of photobiomodulation. Photochem Photobiol Sci. (2018) 17(7):1003–17. 10.1039/C7PP00312A 30044464 PMC6091542

